# Silencing of the Long Non-Coding RNA TTN-AS1 Attenuates the Malignant Progression of Osteosarcoma Cells by Regulating the miR-16-1-3p/TFAP4 Axis

**DOI:** 10.3389/fonc.2021.652835

**Published:** 2021-06-01

**Authors:** Xianghai Meng, Zhenjun Zhang, Lin Chen, Xi Wang, Qingguo Zhang, Shuheng Liu

**Affiliations:** ^1^ Trauma Center, Jinan Central Hospital, Cheeloo College of Medicine, Shandong University, Jinan, China; ^2^ Department of Burn Reconstructive Surgery, Jinan Central Hospital, Cheeloo College of Medicine, Shandong University, Jinan, China; ^3^ Department of Spine Surgery, Jinan Central Hospital, Cheeloo College of Medicine, Shandong University, Jinan, China

**Keywords:** osteosarcoma, long non-coding RNA TTN-AS1, microRNA-16-1-3p, transcription factor activating enhancer binding protein 4, proliferation

## Abstract

**Objectives:**

Osteosarcoma (OS) is a type of bone malignancy. This study attempted to explore the effect of long non-coding RNA TTN-AS1 (TTN-AS1) on OS and to determine its molecular mechanisms.

**Methods:**

The expression of TTN-AS1, microRNA-16-1-3p (miR-16-1-3p), and transcription factor activating enhancer binding protein 4 (TFAP4) in OS was assessed using qRT-PCR. The OS cell proliferation, migration, and invasion were measured using 3-(4,5-Dimethylthiazol-2-yl)-2,5-diphenyltetrazolium bromide (MTT), wound-healing, and transwell assays. N-cadherin and MMP-2 protein level was determined with western blot. Interactions between TTN-AS1 and miR-16-1-3p or TFAP4 and miR-16-1-3p were confirmed using the dual-luciferase reporter assay. Additionally, an OS xenograft tumor model was constructed to assess the effect of TTN-AS1 on tumor growth.

**Results:**

TTN-AS1 and TFAP4 expression was increased in OS, while miR-16-1-3p expression was decreased. TTN-AS1 silencing restrained OS cell proliferation, migration, invasion, N-cadherin and MMP-2 protein expression, and hindered tumor growth. MiR-16-1-3p overexpression retarded the malignant behavior of OS cells. TTN-AS1 played a carcinostatic role by down-regulating miR-16-1-3p in the OS cells. Moreover, miR-16-1-3p inhibition or TFAP4 elevation weakened the suppressive effect of TTN-AS1 silencing on OS cell tumor progression.

**Conclusion:**

TTN-AS1 promoted the proliferation, migration, invasion, and epithelial-mesenchymal transition (EMT) of OS cells *via* mediating the miR-16-1-3p/TFAP4 axis. TTN-AS1 may be a critical target for improving OS.

## Introduction

Osteosarcoma (OS) is a malignancy of the bone that generally occurs in adolescents and children (1). It is characterized by a high level of complexity and heterogeneity (2). Resection surgery, combined with polychemotherapy, is an important therapeutic approach for its treatment (3). The metastasis rate of OS is over 30%, with high recurrence and poor efficacy (4). OS progression is complex and is caused by aberrant oncogenes or anti-oncogenes expression (5, 6). Therefore, it is imperative to investigate the pivotal molecules involved in the malignant progression of OS.

Long non-coding RNAs (lncRNAs) participate in different types of cancers (7–9). Dysregulation of lncRNAs has been uncovered in diverse cancers, including glioma (10), gallbladder cancer (11), and OS (12). LncRNA TTN-AS1 (TTN-AS1) serves as an oncogene in various cancers. For instances, TTN-AS1 interacts with miR-4677-3p to promote lung adenocarcinoma cell proliferation by targeting ZEB1 (13). TTN-AS1 promotes tumor progression of glioblastoma cells by modulating miR-320b expression (14). TTN-AS1 silencing modulates miR-376a-3p to prevent colorectal cancer tumorigenicity (15). Importantly, Li et al. have pointed out that TTN-AS1 expression is up-regulated in OS and overexpression of TTN-AS1 promotes the development of OS (16). Fu et al. have determined that TTN-AS1 regulates OS cell apoptosis and drug resistance (17). Therefore, TTN-AS1 may facilitate OS development. Nevertheless, the underlying mechanism of TTN-AS1 in OS has not yet been elucidated.

It has been documented that microRNAs (miRNAs) exert tumor-suppressive activity in different human malignancies (18, 19). LncRNAs can interact with miRNAs to block miRNA-mediated target gene in OS. For instances, lncRNA DICER1-AS1 accelerates the growth of OS cells through inhibiting miR-30b, which subsequently enhances ATG5 expression (20). LncRNA SNHG5 interacts with miR-212-3p to increase SGK3 expression and promote OS cell growth and metastasis (21). MiR-16-1-3p frequently serves as anti-oncogene. MiR-16-1-3p mediates TWIST1 to inhibit the malignant potential of gastric cancer cells (22). Notably, up-regulation of miR-16-1-3p represses OS cell survival and promotes cell apoptosis (23). Therefore, we investigated whether TTN-AS1 could directly target miR-16-1-3p to regulate the progression of OS.

Transcription factor activating enhancer binding protein 4 (TFAP4) is involved in cancer cell proliferation, epithelial-mesenchymal transition (EMT), stemness, apoptosis, and cellular senescence (24). Thereby, TFAP4 is a critical mediator of the onset and development of cancers (25, 26). TFAP4 overexpression facilitates the malignant behaviors of gastric cancer cells (27). High TFAP4 expression predicts poor outcomes in colorectal cancer patients (28). LncRNA LINC00520 sponges miR-520f-3p by targeting TFAP4 to promote the progression of glioma malignancy (29). However, the regulatory mechanism underlying the association of TTN-AS1 with the miR-16-1-3p/TFAP4 axis in OS is unclear.

Herein, we demonstrated the role of TTN-AS1 in the tumor progression of OS. This study confirmed the relationships among TTN-AS1, miR-16-1-3p, and TFAP4, thus providing a new direction for OS treatment.

## Materials and Methods

### Tissue Samples

Sixty-three patients with OS were recruited from our hospital between April 2016 and January 2018. OS tissues and adjacent tissues were harvested. Patients had never received anti-cancer treatment. This study was permitted by ethics committee of our hospital in accordance with the Declaration of Helsinki, and informed consent was obtained from each patient and guardian.

### Cell Culture

HOS, MG63, and U2OS OS cell lines, and fetal osteoblastic cell line, hFOB, were gained from the American Type Culture Collection (ATCC, Manassas, VA, USA). All cells were cultured in the Dulbecco’s modified Eagle’s medium (DMEM, Invitrogen, Carlsbad, CA, USA), supplemented with 10% fetal bovine serum (FBS, Invitrogen) and 1% penicillin-streptomycin solution (Invitrogen), at 5% CO_2_ and 37°C.

### Cell Transfection

MiR-16-1-3p mimics, miR-16-1-3p inhibitor, miR-negative control (NC), inhibitor NC, pcDNA-NC, and pcDNA-TFAP4 were obtained from GenePharma (Shanghai, China). After reaching 80% confluence, the OS cells were transfected or co-transfected with the aforementioned agents using Lipofectamine 3000 (Invitrogen).

### Plasmid Construction and Lentivirus Production

Short hairpin (sh)-TTN-AS1-1, 2, 3, and sh-NC were prepared and synthesized by GenePharma. MG63 and U2OS cells were infected with harvested sh-TTN-AS1-1, 2, 3 or sh-NC lentivirus supernatants for 48 h and screened with puromycin for at least 7 d, and the sh-TTN-AS1-1, 2, 3 or sh-NC stably transfected cells were obtained.

### Quantitative Real-Time Polymerase Chain Reaction (qRT-PCR)

Total RNA was extracted from tissues and cells using the TRIzol reagent (Invitrogen). cDNA samples were obtained by reverse transcription using the PrimeScript RT Reagent kit (Takara, Tokyo, Japan). The QuantiTect SYBR Green PCR kit (Takara) was used for qRT-PCR analysis. qRT-PCR was performed using a 7500 Real-time PCR system (Applied Biosystems, Foster City, CA, USA) with the following reaction conditions: initial denaturation at 95°C for 10 min, followed by 40 cycles at 95°C for 10 s, 60°C for 20 s, and 72°C for 34 s. The relative expression was calculated using the 2^-ΔΔCq^ method. GAPDH, U6, and β-actin were used to normalize TTN-AS1, miR-16-1-3p, and TFAP4, respectively. Primer sequences are listed in **Table 1**.

**Table 1:** Primers sequences.

Name of primer	Sequences (5’-3’)
TTN-AS1-F	CCAGACACCTAACCAACTTCC
TTN-AS1-R	GTGATCTCATCCCTCTTGCTT
GAPDH-F	AGAAGGCTGGGGCTCATTTG
GAPDH-R	AGGGGCCATCCACAGTCTTC
miR-16-1-3p-F	GGGGCCAGTATTAACTGT
miR-16-1-3p-R	TGCGTGTCGTGGAGTC
U6-F	GCTTCGGCAGCACATATACTAAAAT
U6-R	CGCTTCACGAATTTGCGTGTCAT
TFAP4-F	GCAGGCAATCCAGCACAT
TFAP4-R	GGAGGCGGTGTCAGAGGT
β-actin-F	AAAGACCTGTACGCCAACAC
β-actin-R	GTCATACTCCTGCTTGCTGAT

### Western Blot

Total protein was extracted from tissues and cells using RIPA lysis buffer (Beyotime, Shanghai, China). The concentration of total protein was determined using the bicinchoninic acid method. The proteins were then separated using sodium dodecyl sulphate-polyacrylamide gel electrophoresis. Separated proteins were transferred onto polyvinylidene fluoride membranes, blocked with 5% skim milk for 1 h at 37°C, and incubated at 4°C overnight with primary antibodies, including anti-TFAP4 (1:1000, SAB1412283, Sigma, St. Louis, MO, USA), N-cadherin (1:5000, SAB2702400, Sigma), MMP2 (1:1000, SAB4501891, Sigma), and β-actin (1:4000, SAB2701711, Sigma) antibodies. Thereafter, the membranes were incubated with a horseradish peroxidase-labelled goat anti-rabbit IgG (1:5,000, 12-349MSDS, Sigma) secondary antibody at 25°C for 1 h. The immunoblots were analyzed by enhanced chemiluminescence and semi-quantified using the ImageLab software (Bio-Rad, Hercules, CA, USA).

### 3-(4,5-Dimethylthiazol-2-yl)-2,5-Diphenyltetrazolium Bromide (MTT) Assay

The OS cells (2 × 10^3^ cells/well) were seeded into 96-well plates and cultured with 5% CO_2_ at 37°C. Cell proliferation was obtained by MTT cell proliferation assay kit (Sigma).

### Wound Healing Assay

The OS cells (5 × 10^5^ cells/well) were incubated in 6-well plates until reached about 100% confluence. Then, the cell monolayer was wounded with a pipette tip and the plates were washed twice with phosphate-buffered saline (PBS) to remove detached cells. The cells were then cultured in serum-free DMEM (Invitrogen) at 37°C. During the following 24 h, the cells migrated into the wound area. Cell migration images were captured using an inverted microscope (Olympus, Tokyo, Japan).

### Transwell Assay

The OS cells (2 × 10^5^) in serum-free DMEM (Invitrogen) were added to the upper chambers pre-coated with matrigel (Sigma). DMEM, containing 10% FBS, was added to the lower chambers. Cells remaining in upper chamber were wiped with cotton swab after incubation for 48 h. Cells in lower chambers were fixed with 4% paraformaldehyde for 30 min and stained with 0.1% crystal violet for 30 min. Finally, the images were counted using an inverted microscope (Olympus).

### Tumor Formation

Total 12 male BALB/c nude mice were gained from HFK Bioscience Co., Ltd (Beijing, China). The mice were housed at 22-26°C in an atmosphere of 55-65% relative humidity and fed a normal diet. MG63 cells (1 × 10^6^ cells) expressing sh-NC or sh-TTN-AS1-1 were subcutaneously injected into the flank region of nude mice to establish OS xenograft model (n = 6 per group). Tumor volumes (0.5 × length × width^2^) were assessed on a weekly basis. Four weeks after injection, mice were anesthetized (pentobarbital sodium, 50 mg/kg), sacrificed through cervical dislocation. Finally, intact tumors were exfoliated and weighted. All the animal experiments were approved by the Animal Care and Use Committee of our hospital.

### RNA Immunoprecipitation (RIP) Assay

RIP assay was performed using an EZ-Magna RIP kit (Millipore), following the manufacturer’s instructions to further confirm the target relationship between TTN-AS1 and miR-16-1-3p. OS cells at 85% confluence were lysed in RIP lysis buffer, then the cell extract was incubated with magnetic beads and an antibody against argonaute 2 (Anti-AGO2; ab32381; Abcam, Cambridge, MA, USA) or immunoglobin G (Anti-IgG; ab109761; Abcam) at 4°C overnight. Proteinase K was used to digest the protein and immunoprecipitated RNAs were isolated. Finally, purified RNAs were subject to qRT-PCR analysis.

### Bioinformatics-Based Prediction and Analyses

We used predicting tool, LncBase, for prediction of the miRNAs that target TTN-AS1. MiR-16-1-3p was predicted as one of the candidate miRNAs. MiR-16-1-3p possesses strong tumor suppressive and anti-metastatic properties in OS (23). However, the detail regulatory mechanism between TTN-AS1 and miR-16-1-3p in OS remains unknown. We therefore selected miR-16-1-3p for subsequent studies. We additionally employed the online tool, targetScan, for predicting the possible target genes of miR-16-1-3p. TFAP4 was predicted as one of the candidate mRNAs. TFAP4 is a well-known oncogene, which up-regulated in various cancers (27, 28, 30). However, the detail regulatory mechanism between miR-16-1-3p and TFAP4 in OS remains unknown. We therefore selected TFAP4 for subsequent studies.

### Dual-Luciferase Reporter Assay

TTN-AS1 and TFAP4 with WT or MUT miR-16-1-3p-binding sites were fused to the psiCHECK-2 vectors (Promega, Madison, WI, USA). OS cells were subsequently co-transfected with the aforementioned luciferase vectors and miR-16-1-3p mimics or miR-NC by incubating with Lipofectamine 3000 for 48 h, and detected using the dual-luciferase reporter assay system (Promega).

### Statistical Analysis

All statistical analyses were performed using GraphPad Prism 7.0 (GraphPad Software, La Jolla, CA, USA). Data are presented as mean ± standard deviation (SD). The differences between two groups or among multiple groups were analyzed using the Student’s t-test or one-way ANOVA followed by Tukey’s post-hoc test. The significance of the correlations was determined using Pearson’s correlation analysis. P values < 0.05 were considered as statistically significant.

## Results

### TTN-AS1 Expression Was Enhanced in Human OS Tissues

**Figure 1:**
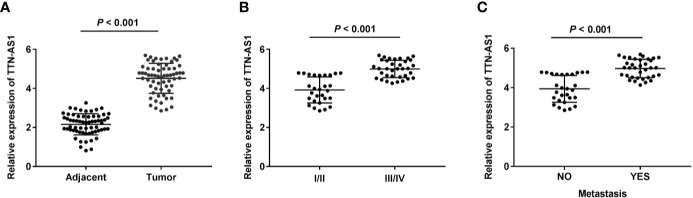
TTN-AS1 expression was increased in human osteosarcoma (OS). **(A)** TTN-AS1 expression in tumor and adjacent tissues was measured by qRT-PCR. Data were analyzed by paired Student’s t-test; **(B)** TTN-AS1 expression in OS patients at TNM I/II and TNM III/IV was detected by qRT-PCR. Data were analyzed by unpaired Student’s t-test; **(C)** TTN-AS1 expression in tumors with and without metastasis was tested by qRT-PCR. Data were analyzed by unpaired Student’s t-test.

qRT-PCR was performed to confirm whether TTN-AS1 was differentially expressed in OS tumor. Results displayed that TTN-AS1 expression was dramatically enhanced in tumor tissues of OS patients (P < 0.001, **Figure 1A**). Additionally, TTN-AS1 expression was higher in tissues from patients in TNM III/IV (P < 0.001, **Figure 1B**). TTN-AS1 expression was significantly up-regulated in the metastatic tumors (P < 0.001, **Figure 1C**). As displayed in **Table 2**, TTN-AS1 expression was positively correlated with the degree of metastasis (P < 0.05), and WHO grade (P < 0.01) in OS patients.

**Table 2:** Correlation between TTN-AS1 expression and clinicopathological features in osteosarcoma patients.

Characteristics	n	TTN-AS1(Low) 31	(High) 32	P value
Age				0.716
<18 years	36	17	19	
≥18 years	27	14	13	
Gender				0.868
Male	23	11	12	
Females	40	20	20	
Tumor Size				0.367
<8cm	28	12	16	
≥8cm	35	19	16	
Histological subtype				0.712
Osteoblastic	34	16	18	
Fibroblastic	29	15	14	
Metastasis				0.032^*^
No	28	18	10	
Yes	35	13	22	
WHO Grade				0.008^**^
I+II	28	19	9	
III+IV	35	12	23	

^*^P < 0.05, ^**^P < 0.01. WHO, World Health Organization.

### TTN-AS1 Knockdown Repressed the Progression of OS

**Figure 2:**
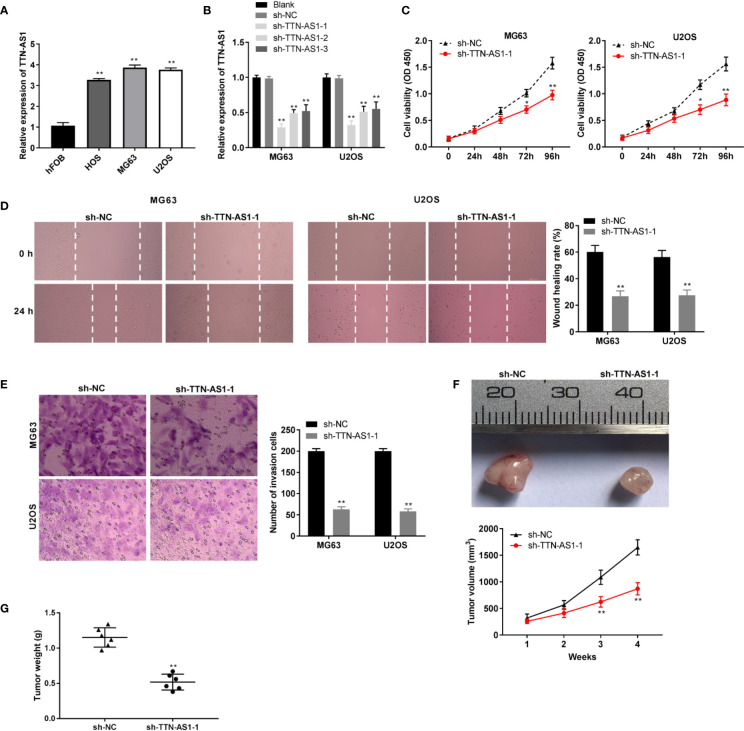
TTN-AS1 down-regulation repressed osteosarcoma (OS) progression **(A)** TTN-AS1 expression in hFOB, HOS, MG63, and U2OS cells was evaluated by qRT-PCR. ^**^P < 0.01 *vs*. hFOB. Statistical analysis was conducted using one-way ANOVA followed by Tukey’s post-hoc test; **(B)** Transfection efficiency of sh-NC and sh-TTN-AS1-1, -2, and -3 was demonstrated using qRT-PCR. Statistical analysis was conducted using one-way ANOVA followed by Tukey’s *post-hoc* test; **(C)** MG63 and U2OS cell proliferation were measured by MTT assay. Data were analyzed by unpaired Student’s t-test; **(D)** Wound healing assay was conducted to test the wound healing rate of MG63 and U2OS cells. Data were analyzed by unpaired Student’s t-test; **(E)** Number of invasion cells was determined by transwell assay and the invasion rate was calculated. Data were analyzed by unpaired Student’s t-test; The effects of TTN-AS1 suppression on tumor volume **(F)** and tumor weight **(G)** are shown. ^*^P < 0.05, ^**^P < 0.01 *vs*. sh-NC. Data were analyzed by unpaired Student’s t-test.

We used RNA interference approaches to investigate whether knockdown of TTN-AS1 affects the progression of OS. TTN-AS1 expression was higher in the HOS, MG63, and U2OS cells than that in the hFOB cells (P < 0.01, **Figure 2A**). MG63 and U2OS cells (OS cells) were selected for subsequent experiments because of their high TTN-AS1 expression. TTN-AS1 expression was markedly decreased by the transfection of sh-TTN-AS1-1, -2, and -3 into OS cells (P < 0.01, **Figure 2B**). Sh-TTN-AS1-1 was used for subsequent experiments due to its relatively high silence efficiency. Subsequently, sh-TTN-AS1-1 considerably decreased OS cell proliferation after 72 (P < 0.05) and 96 h (P < 0.01, **Figure 2C**) of culture. Moreover, TTN-AS1 down-regulation notably reduced invasion and migration of OS cells (P < 0.01, **Figures 2D, E**). TTN-AS1 silencing markedly reduced the tumor volume and weight in mice (P < 0.05, **Figures 2F, G**).

### TTN-AS1 Conversely Modulated miR-16-1-3p Expression in OS

**Figure 3:**
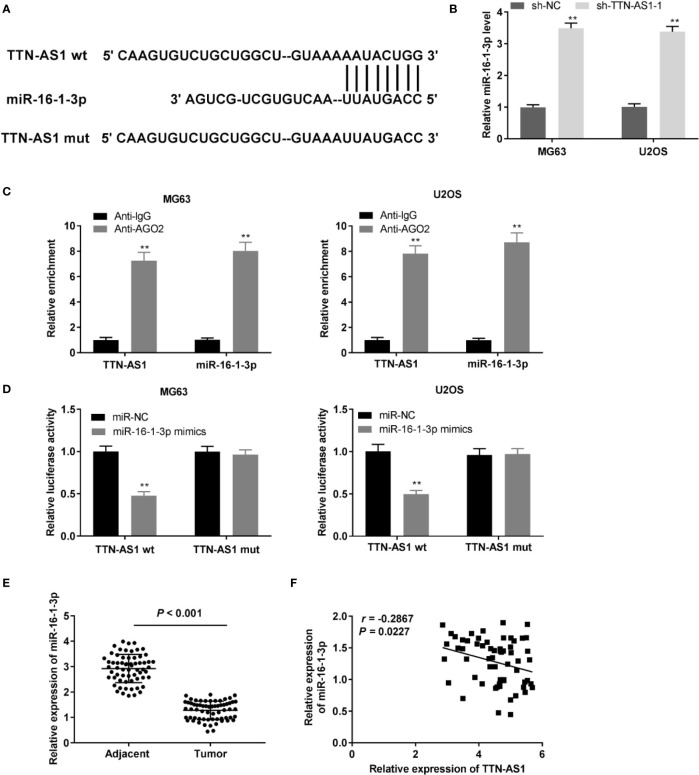
TTN-AS1 conversely modulated miR-16-1-3p. **(A)** LncBase illustrated the binding site between TTN-AS1 and miR-16-1-3p; **(B)** MiR-16-1-3p expression in MG63 and U2OS cells was assessed by qRT-PCR. ^**^P < 0.01 *vs*. sh-NC. Data were analyzed by unpaired Student’s t-test; **(C)** RIP assay was conducted to confirm the enrichment degrees of TTN-AS1 and miR-16-1-3p in IgG or AGO2 immunoprecipitate. ^**^P < 0.01 *vs*. Anti-IgG. Data were analyzed by unpaired Student’s t-test; **(D)** Luciferase activity in MG63 and U2OS cells was evaluated. ^**^P < 0.01 *vs*. miR-NC. Firefly luciferase activity was normalized to Renilla luciferase activity. Data were analyzed by unpaired Student’s t-test; **(E)** QRT-PCR was performed to detect the miR-16-1-3p expression in tumor and adjacent tissues. Data were analyzed by paired Student’s t-test; **(F)** TTN-AS1 expression was negatively correlated with miR-16-1-3p. Correlation significance was evaluated by Pearson correlation analysis.

We identified miR-16-1-3p as a potential target of TTN-AS1 through LncBase (**Figure 3A**). TTN-AS1 knockdown markedly enhanced miR-16-1-3p expression in OS cells (P < 0.01, **Figure 3B**). Then, the RIP assay revealed that both TTN-AS1 and miR-16-1-3p were abundant in AGO2 RIP of OS cells compared with IgG RIP (P < 0.01, **Figure 3C**). Next, miR-16-1-3p mimics reintroduction considerably decreased the luciferase activity in OS cells transfected with TTN-AS1 Wt compared with miR-NC (P < 0.01, **Figure 3D**). MiR-16-1-3p expression was considerably reduced in tumor tissues compared to adjacent tissues in OS patients (P < 0.001, **Figure 3E**). Furthermore, a converse correlation between TTN-AS1 and miR-16-1-3p expression was observed in OS tissues (r = -0.2867, P = 0.0227, **Figure 3F**).

### MiR-16-1-3p Inhibited the Tumor Progression of OS Cells

**Figure 4:**
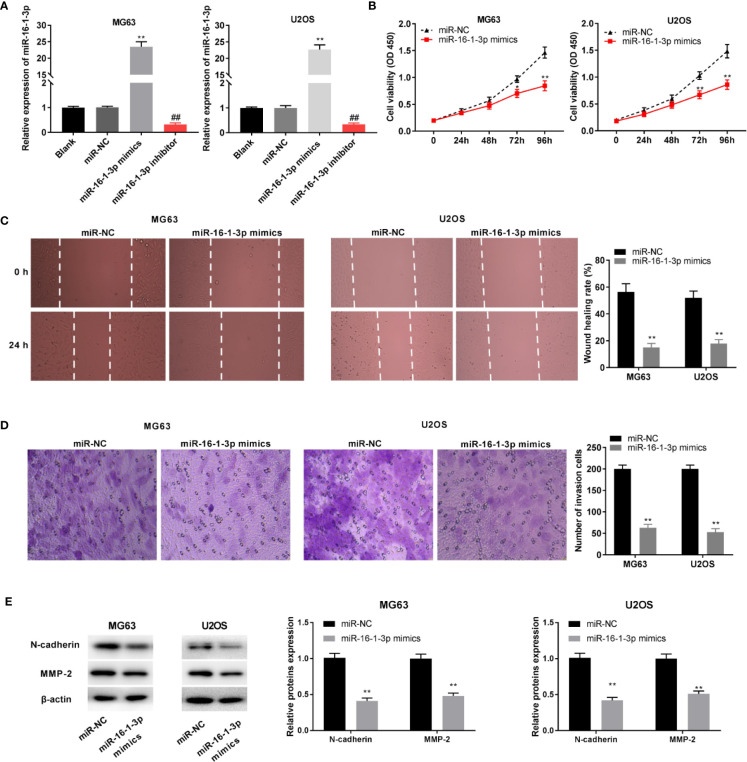
MiR-16-1-3p constrained tumor progression of osteosarcoma (OS) cells. **(A)** QRT-PCR was performed to evaluate transfection efficiency of miR-NC, miR-16-1-3p mimics, and miR-16-1-3p inhibitor. ^**^P < 0.01, ##P < 0.01 *vs*. miR-NC. Statistical analysis was conducted using one-way ANOVA followed by Tukey’s *post-hoc* test; MTT assay **(B)**, wound healing assay **(C)**, transwell assay **(D)**, and western blot **(E)** were conducted following miR-16-1-3p elevation. ^*^P < 0.05, ^**^P < 0.01 *vs*. miR-NC. Data were analyzed by paired Student’s t-test.

MiR-16-1-3p expression was elevated and antagonized by the transfection of miR-16-1-3p mimics and miR-16-1-3p inhibitor into OS cells (P < 0.01, **Figure 4A**). MiR-16-1-3p considerably decreased OS cell proliferation after 72 (P < 0.05) and 96 h of culture (P < 0.01, **Figure 4B**). Migration and invasion of OS cells were decreased by miR-16-1-3p overexpression (P < 0.01, **Figures 4C, D**). Next, the protein expression of N-cadherin and MMP-2 was measured with western blot. MiR-16-1-3p mimics clearly inhibited EMT-related protein, N-cadherin in OS cells (P < 0.01). Following miR-16-1-3p overexpression, the protein expression of MMP2, which plays key roles in the cell migration/invasion, was markedly decreased in OS cells (P < 0.01, **Figure 4E**).

### TFAP4 Was a Target of miR-16-1-3p

**Figure 5:**
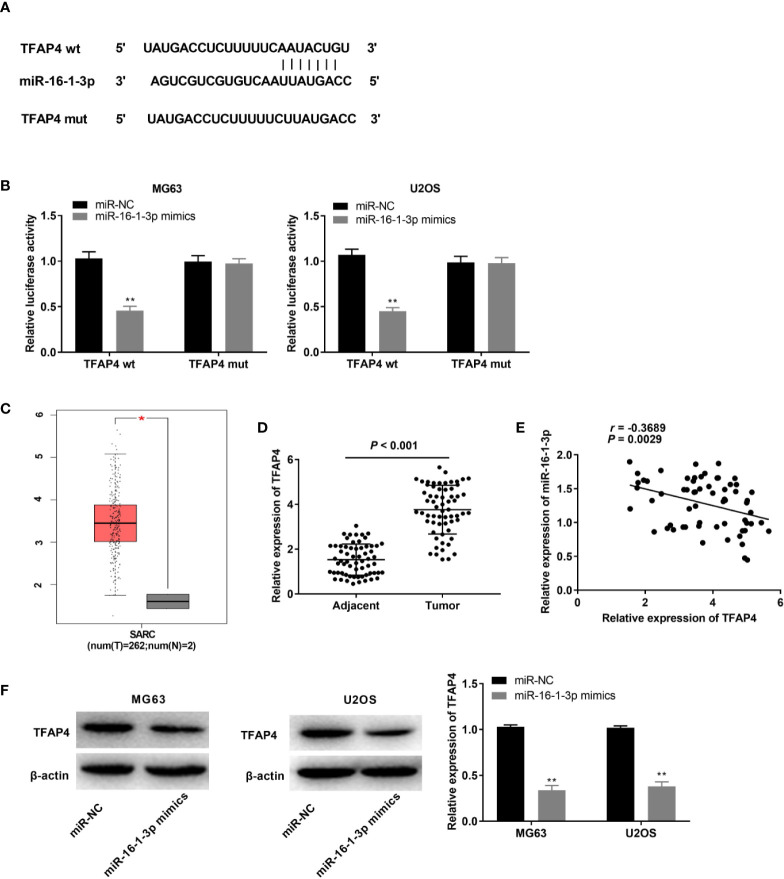
MiR-16-1-3p targeted by TFAP4. **(A)** TargetScan illustrated the binding site between TFAP4 and miR-16-1-3p; **(B)** Luciferase activity in MG63 and U2OS cells was assessed. ^**^P < 0.01 *vs*. miR-NC. Data were analyzed by unpaired Student’s t-test; **(C)** TFAP4 expression in OS tissues and normal bone tissues was analyzed by GEPIA. *P < 0.05 vs. normal tissues. **(D)** QRT-PCR was used to obtain TFAP4 expression in adjacent tissues and tumor tissues in OS patients. Data were analyzed by paired Student’s t-test; **(E)** TFAP4 expression was negatively correlated with miR-16-1-3p. Correlation significance was evaluated by Pearson correlation analysis; **(F)** TFAP4 protein expression was obtained by western blot. ^**^P < 0.01 *vs*. miR-NC. Data were analyzed by unpaired Student’s t-test.

We used TargetScan to predict a binding site for miR-16-1-3p on the 3’ UTR of TFAP4 (**Figure 5A**). MiR-16-1-3p up-regulation markedly repressed the luciferase activity of the WT TFAP4 reporter vector in OS cells (P < 0.01, **Figure 5B**). TFAP4 expression in 2 normal osteoblast tissues was lower than those in 262 OS tissues, as assessed with GEPIA. The results displayed that TFAP4 expression was higher in the OS tissues than that in the normal osteoblast tissues (P < 0.05, **Figure 5C**). TFAP4 expression was considerably enhanced in tumor tissues of OS patients (P < 0.001, **Figure 5D**), and was inversely correlated with miR-16-1-3p expression in OS tissues (r = -0.3689, P = 0.0029, **Figure 5E**). Additionally, transfection of miR-16-1-3p mimics hindered TFAP4 expression in OS cells (P < 0.01, **Figure 5F**).

### TTN-AS1 Silencing Impeded OS Cell Tumor Progression by Mediating the miR-16-1-3p/TFAP4 Axis

**Figure 6:**
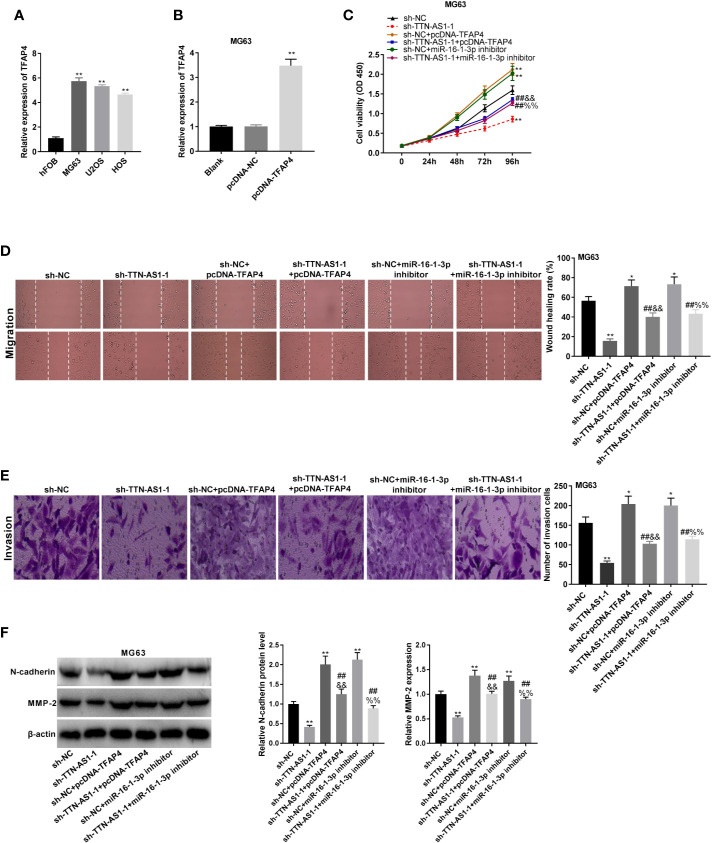
TTN-AS1 silencing impeded osteosarcoma (OS) cell tumor progression by regulating the miR-16-1-3p/TFAP4 axis. **(A)** TFAP4 expression in hFOB, MG63, U2OS, and HOS cells was obtained by qRT-PCR. ^**^P < 0.01 *vs*. hFOB. Statistical analysis was conducted using one-way ANOVA followed by Tukey’s *post-hoc test* test; **(B)** QRT-PCR was performed to detect the transfection efficiency of pcDNA-NC and pcDNA-TFAP4 in MG63 cells. ^**^P < 0.01 *vs*. pcDNA-NC. Statistical analysis was conducted using one-way ANOVA followed by Tukey’s *post-hoc test* test; MiR-16-1-3p elevation or TFAP4 inhibition mitigated the inhibitory effects of TTN-AS1 deficiency on proliferation **(C)**, migration **(D)**, and invasion **(E)** of MG63 cells. ^*^P < 0.05, ^**^P < 0.01 *vs*. sh-NC; ^##^P < 0.01 *vs*. sh-TTN-AS1-1; ^&&^P < 0.01 *vs*. sh-NC+pcDNA-TFAP4; ^%%^P < 0.01 *vs*. sh-NC+miR-16-1-3p inhibitor. Statistical analysis was conducted using one-way ANOVA followed by Tukey’s *post-hoc test* test; **(F)** MiR-16-1-3p overexpression or TFAP4 knockdown rescued the suppressed protein expression of N-cadherin and MMP2 caused by TTN-AS1 depletion in MG63 cells. ^**^P < 0.01 *vs*. sh-NC; ^##^P < 0.01 *vs*. sh-TTN-AS1-1; ^&&^P < 0.01 *vs*. sh-NC+pcDNA-TFAP4; ^%%^P < 0.01 *vs*. sh-NC+miR-16-1-3p inhibitor. Statistical analysis was conducted using one-way ANOVA followed by Tukey’s *post-hoc test*.

TFAP4 expression was dramatically elevated in MG63, U2OS, and HOS cells (P < 0.01, **Figure 6A**). MG63 cells were selected for subsequent experiments because of their high TFAP4 expression. TFAP4 expression was up-regulated by the transfection of pcDNA-TFAP4 into MG63 cells (P < 0.01, **Figure 6B**). To demonstrate whether TTN-AS1 knockdown attenuates OS development by modulating miR-16-1-3p and TFAP4, we performed rescue experiments in OS cells. As displayed in **Figures 6C–E**, the proliferation, migration, and invasion of MG63 cells were significantly decreased by TTN-AS1 inhibition (P < 0.01), but were increased by TFAP4 overexpression or miR-16-1-3p inhibition (P < 0.05). The proliferation, migration, and invasion of MG63 cells were lower in the sh-TTN-AS1-1 + pcDNA-TFAP4 group than those in the sh-NC + pcDNA-TFAP4 group (P < 0.01), and were lower in the sh-TTN-AS1-1 + miR-16-1-3p inhibitor group than those in the sh-NC + miR-16-1-3p inhibitor group (P < 0.01). TFAP4 overexpression or miR-16-1-3p silencing visibly reversed the inhibitory effect of sh-TTN-AS1-1 on tumor progression of MG63 cells (P < 0.01). As displayed in **Figure 6F**, the protein expression of N-cadherin and MMP2 protein was decreased by TTN-AS1 inhibition (P < 0.01), but were increased by TFAP4 overexpression or miR-16-1-3p inhibition (P < 0.01). The protein expression of N-cadherin and MMP2 protein was lower in the sh-TTN-AS1-1 + pcDNA-TFAP4 group than those in the sh-NC + pcDNA-TFAP4 group (P < 0.01), and was lower in the sh-TTN-AS1-1 + miR-16-1-3p inhibitor group than those in the sh-NC + miR-16-1-3p inhibitor group (P < 0.01). TFAP4 overexpression or miR-16-1-3p silencing markedly rescued the suppression of N-cadherin and MMP2 protein expression caused by TTN-AS1 knockdown in MG63 cells (P < 0.01).

## Discussion

Up-regulation of lncRNAs, such as lncRNA BCAR4 (31), lncRNA TUG1 (32), and lncRNA MF12 (33) has been shown to exert a pivotal influence on OS pathogenesis. Here, TTN-AS1 expression was increased in tumor tissues of OS patients, and was associated with WHO grade and metastasis in OS patients. The function of TTN-AS1 was found to be similar to that of some lncRNAs in OS. The enhanced expression of lncRNA TP73-AS1 is related to distant metastasis and predicts poor outcome in OS patients (34). LncRNA MALAT1 is overexpressed in OS and is clearly correlated with distant metastasis and advanced clinical stage (35). Above all, we suggested that TTN-AS1 expression may be related to OS progression.

TTN-AS1 acts as an oncogene in various malignancies. TTN-AS1 sponges miR-142-5p to modulate CDK5, triggering the growth and metastasis of lung adenocarcinoma (36). TTN-AS1 mediates miR-153-3p to accelerate malignant behaviors by mediating ZNRF2 in papillary thyroid cancer (37). TTN-AS1 suppression hinders the progression of ovarian cancer by up-regulating miR-139-5p (38). Notably, TTN-AS1 binds with miR-134-5p to increase MBTD1 expression, elevate OS cell viability, and inhibit cell apoptosis (17). In this study, TTN-AS1 silencing restrained the OS cell tumor progression. Additionally, some lncRNAs knockdown suppressed tumor growth in OS. For examples, inhibition of lncRNA TUG1 elevates miR-9-5p expression and attenuates the growth of OS tumor xenografts (39). Knockdown of lncRNA miR210HG reduces the OS tumor volume and weight in mice (40). LncRNA TAB silencing suppresses the OS tumor growth *in vivo* (41). Similarly, in our study, TTN-AS1 silencing retarded the OS tumor growth, thereby corroborating the anticarcinogenic effect of TTN-AS1 silencing on OS tumor progression *in vivo*. However, the TTN-AS1 expression is not correlated with tumor size in human. This finding is similar with several previous studies on other LncRNAs and miRNAs in OS, such as LncRNA CBR3-AS1 (42), LncRNA BE503655 (43), and miRNA-223 (44). This phenomenon may be attributed to that the size of primary OS in human can be influenced by massive factors *in vivo*, and the detailed mechanisms leading to this situation still needs to be studied.

An increasing number of studies have shown that lncRNAs influence tumor progression by working as sponges or competing endogenous RNAs (ceRNAs) of miRNAs. Interactions between miRNAs and lncRNAs, including lncRNA MALAT1 and miR-205 (45), lncRNA OIP5-AS1 and miR-223 (46), and lncRNA AWPPH and miR-93-3p (47) have been discovered in OS by numerous researches. Here, miR-16-1-3p is a target of TTN-AS1. Previous studies have shown that miR-16-1-3p expression is decreased and plays a key role in tumor development. MiR-16-1-3p retards gastric cancer tumor progression by modulating TWIST1 (22). MiR-16-1-3p is down-regulated and has an anti-tumoral effect in NSCLC (48). Furthermore, miR-16-1-3p exerts vigorous tumor repressive and anti-metastatic effects in OS (23). Here, miR-16-1-3p was negatively correlated to TTN-AS1 in OS. MiR-16-1-3p deficiency eliminated the suppressive effect of sh-TTN-AS1-1 on OS cells. The above-mentioned results demonstrated that TTN-AS1 knockdown attenuates OS cell tumor progression by increasing miR-16-1-3p expression.

TFAP4 is a direct transcriptional target of certain miRNAs and participates in the tumor progression of diverse tumors. For examples, miR-302c attenuates cell EMT and metastasis through decreasing TFAP4 expression in colorectal cancer (49). MiR-608 induces cell apoptosis by targeting TFAP4 in NSCLC (50). LncRNA LINC00520 interacts with miR-520f-3p to promote the malignant behaviors of glioma cells through targeting TFAP4 (29). TFAP4 is up-regulated and serves as an oncogene in various cancers, such as gastric cancer (27), neuroblastoma (25), and hepatocellular carcinoma (51). Here, TFAP4 expression was elevated in OS cells and was inversely correlated with miR-16-1-3p expression. Given the relationship between TTN-AS1 and miR-16-1-3p, we hypothesized that TTN-AS1 knockdown may inhibit TFAP4 by up-regulating miR-16-1-3p in OS cells. Encouragingly, the feedback experiments showed that up-regulation of TFAP4 mitigated the anti-tumor function of sh-TTN-AS1-1 in OS cells. Taken together, we suggest that sh-TTN-AS1-1 exerts its anti-tumor role through mediating miR-16-1-3p/TFAP4 axis in OS. Moreover, previous studies have demonstrated that some other TTN-AS1 related axes were also involved in the regulation of OS. For instances, TTN-AS1 knockdown inhibits OS cell proliferation, migration, and invasion and induces apoptosis *via* targeting the miR-376a/dickkopf-1 axis (16). Down-regulation of TTN-AS1 not only decreases OS cell viability and drug resistance, but also prompts cell apoptosis by regulating the miR-134-5p/MBTD1 axis (17). Above all, we speculated that there are many other downstream targets of TTN-AS1 that have not yet been determined in OS. Related exploration will be considered in our future studies.

Collectively, our results indicated that TTN-AS1 expression was elevated in OS. TTN-AS1 increased TFAP4 expression through competition with miR-16-1-3p, thus playing an oncogenic role in OS. Additionally, TTN-AS1 deficiency attenuated the OS cell proliferation, migration, invasion, and EMT by regulating the miR-16-1-3p/TFAP4 axis. Findings of the current study may provide a promising therapeutic target for OS.

## Data Availability Statement

The original contributions presented in the study are included in the article/supplementary material. Further inquiries can be directed to the corresponding authors.

## Ethics Statement

The studies involving human participants were reviewed and approved by Ethics Committee of Jinan Central Hospital, Cheeloo College of Medicine, Shandong University. Written informed consent to participate in this study was provided by the participants’ legal guardian/next of kin.

## Author Contributions

XM: Substantial contributions to conception and design, data acquisition, drafting the article, and final approval of the version to be published. ZZ and LC: data acquisition and drafting the article. XW and QZ: data acquisition and final approval of the version to be published. SL: Data analysis and interpretation, and drafting the article. All the authors took part in the experiment. All authors contributed to the article and approved the submitted version.

## Conflict of Interest

The authors declare that the research was conducted in the absence of any commercial or financial relationships that could be construed as a potential conflict of interest.
